# Beyond Dosage: The Need for More Realistic Research Scenarios to Understand Pesticide Impacts on Agricultural Soils

**DOI:** 10.1021/acs.jafc.4c12818

**Published:** 2025-04-16

**Authors:** Judith Riedo, Matthias C. Rillig, Florian Walder

**Affiliations:** † Institute of Biology, Freie Universität Berlin, Altensteinstraße 6, 14195 Berlin, Germany; ‡ Agroecology and Environment, Agroscope, Reckenholzstrasse 191, 8046 Zurich, Switzerland

**Keywords:** Pesticide pressure, environmental risk assessment, soil health, application frequency, mixtures, long-term pesticide effects

## Abstract

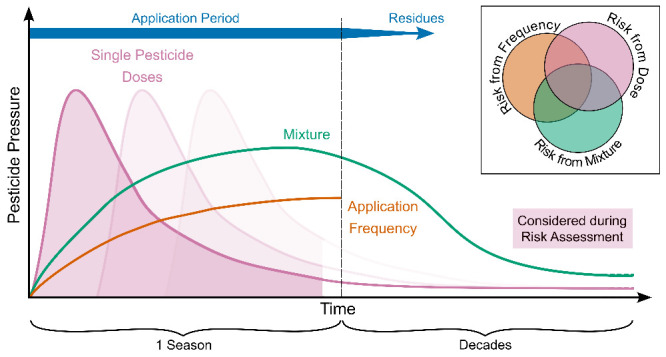

Pesticides play a crucial role in modern agriculture, yet they pose considerable risks to soil health and ecosystem integrity. Current risk assessment research often relies on simplified models, focusing on single substances under standardized conditions and failing to reflect realistic exposure scenarios. We call for a paradigm shift toward incorporating agroecological research that evaluates pesticide effects under more complex and dynamic conditions, including mixtures, application frequency, diverse soil properties, and interactions with other environmental stressors. Additionally, multiseasonal exposure and long-term persistence of pesticides in soils must be considered. By integrating these multidimensional factors, such experimental research can yield valuable insights that improve environmental risk assessment frameworks, ensuring they more accurately represent the complexity of agricultural systems and support more sustainable soil management practices.

## Introduction

In modern agriculture, pesticides are the most effective means of controlling pests, weeds, and diseases, contributing to over 50% of global crop yields.^1,2^ This success has led to unprecedented increases in sales volume and the number of products available,^3^ which have remained consistently high over the past decades.^4^ The desired yield gains come at the cost of unwanted side effects with far-reaching environmental consequences; pesticides have been linked to biodiversity loss, including declines in arthropods, especially beneficial insects, birds, and other wildlife, as well as freshwater contamination.^5−9^ In addition, there is increasing evidence that agricultural soils are particularly exposed and are still widely contaminated decades after the last pesticide application.^10−12^ Consequently, concerns about the potential risks to soil health and associated ecosystem services have come to the forefront.

Pesticides, as bioactive and toxic substances, are tested for their potential risk to nontarget organisms and the environment before commercial use. A pesticide can only be registered and applied on the field after passing these environmental risk assessments. In the European Union (EU), the European Food Safety Authority (EFSA) is responsible for evaluating the environmental risk assessment as part of the pesticide approval process. EFSA evaluates whether the active substances in pesticides pose harmful effects on human and animal health and whether they have unacceptable effects on the environment, including effects on nontarget organisms such as birds, mammals, aquatic organisms, bees, arthropods, flora and soil organisms.^13^ However, pesticide manufacturers carry out the necessary risk assessment studies under strict regulatory guidelines, while EFSA independently reviews the data submitted to ensure that scientific and safety standards are met before making its recommendations.^14^

**Figure 1:**
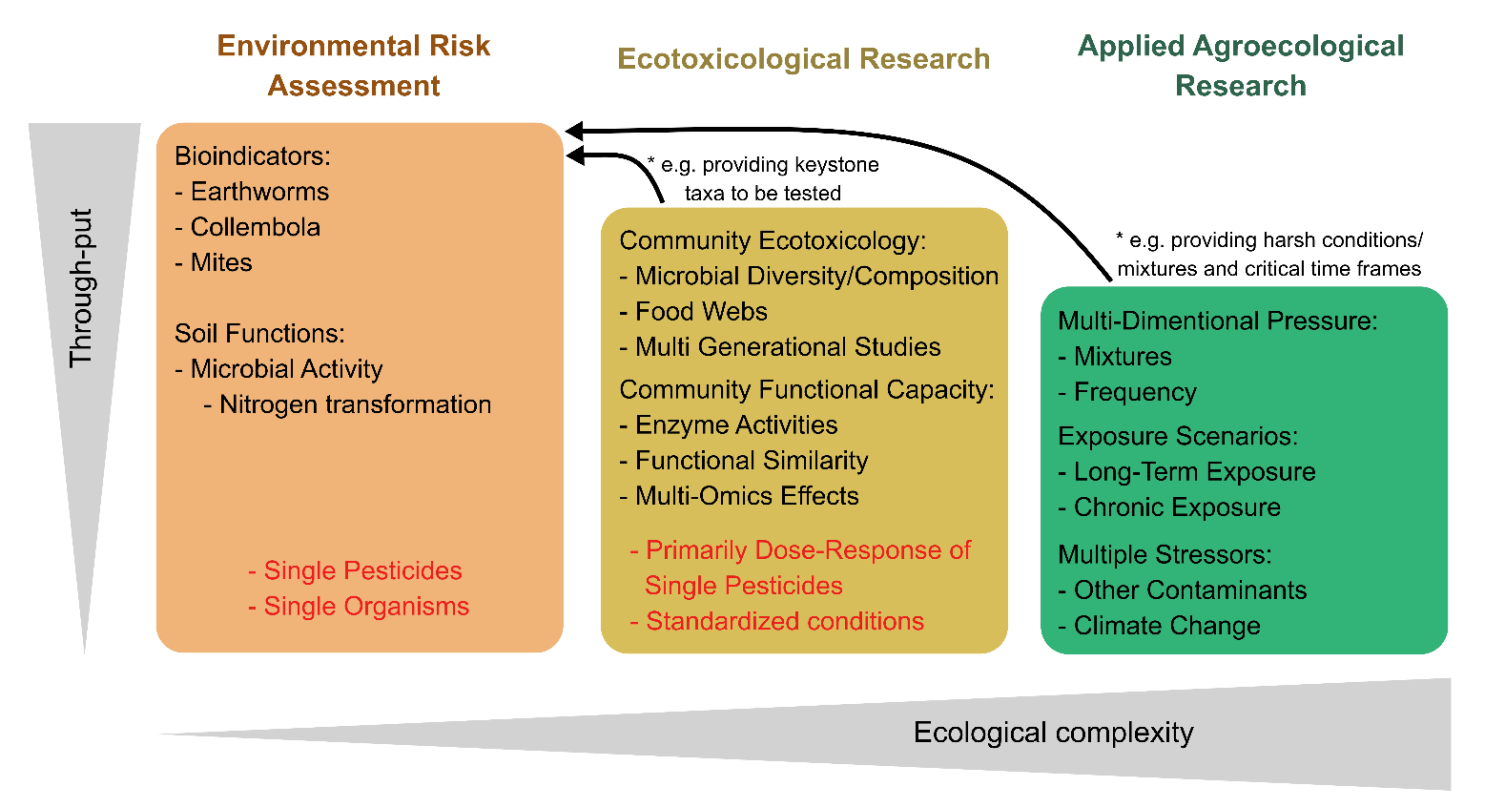
Conceptual framework illustrating the relationship between environmental risk assessment (ERA), ecotoxicological research, and applied agroecological research in assessing the impact of pesticides on soil ecosystems. The figure presents a continuum from standardized high-throughput ERA methods to more complex ecological studies. The leftmost section (orange) represents conventional ERA, which primarily focuses on assessing the effects of individual pesticides on single species (bioindicators) such as earthworms, Collembola, and mites. In this phase, soil function end points, such as nitrogen transformation, are evaluated to estimate pesticide impact. The middle section (yellow) highlights ecotoxicological research, which expands the focus beyond single-species toxicity to encompass community- and ecosystem-level effects. This includes end points such as microbial diversity, enzyme activities, and functional similarity, as well as more complex ecosystem-level assessments like multigenerational studies. However, these studies often still rely on a dose–response approach rather than considering long-term environmental variables. The rightmost section (green) represents experiments accounting for the complexity in the agroecological context, which incorporates realistic pesticide pressure conditions (e.g., mixtures and application frequencies) and exposure scenarios (e.g., long-term or chronic exposure and multiple stressors). Such applied agroecological research provides insight into the cumulative and interactive effects of pesticides under real-world conditions. The figure illustrates a trade-off between throughput and ecological complexity, with ERA focusing on standardized, high-throughput testing, while agroecological research integrates multiple environmental variables to reflect natural conditions more accurately. Arrows indicate how knowledge from ecotoxicological and applied agroecological research feeds back into improving environmental risk assessments.

The impact of pesticides on soils is currently assessed within the frame of the EFSA evaluation using tests on a limited number of individual soil organisms, which serve as bioindicators for key soil processes such as organic matter decomposition and nutrient cycling (Figure 1, left). These tests primarily measure the direct effects of pesticides on the survival, growth and reproduction of these organisms by evaluating the sublethal effects on earthworms over 56 days,^15^ and conducting reproduction tests on Collembola and mites over 14 days.^16−19^ In addition to investigating pesticide effects on individual organisms, the impact on soil microbial communities is assessed by testing the effect on nitrogen mineralization, the conversion of organic to mineral nitrogen by soil microbes, after a minimum of 28 days.^20^ All these assays assess the impact of pesticides under constant and standardized conditions, which are considerably different than real-world agricultural circumstances. Given the simplicity of the test systems, it is not surprising that current risk assessments have limitations in addressing the complexity of soil systems and the potential effects of pesticides on soil health.^21^

Similarly, ecotoxicological research on pesticide effects in soils has traditionally also focused on simplistic systems, testing the effects of individual pesticides on specific soil-dwelling organisms or the biotic communities of one or few soils (Figure 1, center).^22−25^ Such experiments have generated important insights into how pesticides interact with the soil system and how fast and by whom they are degraded.^26−30^ These experimental efforts also revealed the sensitivity of individual species or groups of soil organisms to pesticides. For instance, numerous studies have indicated that microbial processes in the soil nitrogen cycle, particularly the ammonium-oxidizing guilds of bacteria and archaea, are highly sensitive to the exposure of specific pesticides.^25,31−33^ Similarly, soil invertebrates have been predominantly observed to experience adverse effects upon exposure to individual pesticides.^34^ Such experiments have also demonstrated clear effects at the community level, but primarily at the highest, often unrealistic, doses.^34−36^ At recommended dosages, the impact of applying individual pesticides often remains neutral or transient.^35,36^ Remarkably, the results of such experiments become ambiguous when averaged across these ecotoxicological studies.^37^ This is partly due to the wide range of effects observed across different studies, which depend on factors such as the tested compound, the model organism, the physicochemical conditions of the experimental system, and the duration of exposure.^37^ Similarly, in studies testing the effects of individual pesticides at different dosages in higher-tier experiments, the considerable variability inherent to field studies further limits the ability to pinpoint clear effects on soil systems.^21^

In contrast, agroecological studies based on retrospective risk assessment provided robust evidence of pesticides’ adverse effects on soil organisms, irrespective of the compound applied and the pedoclimatic conditions observed (Figure 1, right).^10,38,39^ To close the gaps between agroecological studies and ecotoxicological research, experimental efforts must go beyond assessing the effects of individual substances at different dosages and instead focus on providing a deeper understanding of the cause-and-effect relationships of pesticides in agricultural soils.^40^ This requires addressing the inherently complex nature of pesticide exposure and its effects in agroecosystems, reflecting the use of elaborate spray plans to manage insect pests, weeds and pathogens season after season in agricultural settings. Such spray plans apply pesticides as combination products or tank mixtures at the recommended dosages at different time points across a crop’s growing season, application scenarios that are not yet or not sufficiently considered during risk assessment and seldom integrated in current ecotoxicological research on pesticide effects.^21,41,42^ Hence, the question arises whether these current efforts, using mostly simplified settings, can comprehensively address the pesticide pressure to which agricultural soils are exposed and if they adequately capture the potential risks of pesticides to soil ecosystem services now and in the long term.

In this opinion, we argue that a shift in perspective is needed to assess the risk of pesticide application on soil systems thoroughly. Rather than focusing solely on the toxicity of individual pesticides in controlled settings, research should also adopt an experimental approach that better reflects the complexity of agroecosystems. This includes testing entire soil systems under conditions that more closely reflect actual agricultural practices, including realistic pesticide mixtures, application timing, and cumulative exposure effects as they occur in a spray plan. In addition, extended experimental periods are essential to capture long-term effects, while studies should also account for environmental variability, such as fluctuations in resource availability, temperature, and moisture. By integrating these factors, such prospective agroecological research can generate additional relevant insights that improve our understanding of pesticide effects in dynamic soil environments and provide essential knowledge to refine and improve current risk assessment frameworks.

## The Three Dimensions of Pesticide Pressure in Spray Plans

Tailoring spray plans to the specific needs of crops leads to different dimensions of pesticide pressure on agricultural soils. The pressure in the spray plans is influenced by the total amount of substance applied (the sum of the recommended dosages), the complexity of the mixture (the number of different compounds applied), and the frequency of application (the number of times the substances are applied within a given period). These three key dimensions determine the intensity of pesticide pressure on agricultural soils during a cropping season, which may even amplify each other. Current research on the impact of pesticides on soils has largely focused on dose responses, with growing attention to mixtures, while temporal aspects, such as frequency of application, have not yet been thoroughly investigated, although there is a growing body of evidence pointing to the importance of these factors in determining the environmental impact of pesticides.

### Pesticide Dosage

The role of dosage is an important starting point for discussing the effects of pesticides on our environment, as it is fundamental to assessing the effects of bioactive substances, whether for ecological risk assessment or determining the optimal dosage for controlling weeds, pests, or pathogens. The relationship between a substance dosage and the activity of nontarget organisms, explored in dose–response curves, is employed to estimate the most commonly used indicators for pesticide risks, such as the EC_10_ or EC_20_ (the concentration that results in effects in 10 or 20% of the organisms tested) and the NOEC (the no observed effect concentration, which represents the highest dose or level of exposure to a toxicant that does not produce a noticeable toxic effect on an organism).^43^ Such dose-dependent information is currently the centerpiece for the assessment of potential risks of pesticides to crops, wildlife, and ecosystems, representing the basis for subsequent decisions by regulators on the safe use of pesticides and their potential impact on the environment. Similarly, and as outlined above, much ecotoxicological research has been conducted on the dose-dependent effects of pesticides. The current one-dimensional efforts, while valuable, are designed to evaluate the detailed univariate relationship between particular substances and the impact on specific organisms, including distinguishing between immediate (acute) and prolonged (chronic) impacts. Nevertheless, this approach may not be appropriate for addressing scenarios where numerous substances interact dynamically over time, highlighting the need for a more comprehensive approach.

### Pesticide Mixture

Exposure to diverse pesticide mixtures is the prevailing agronomically relevant situation reflecting pesticide pressure in agroecosystems. The number of different pesticides applied to a field within a cropping season varies widely depending on factors such as crop type, pest pressure, climatic conditions, and farming system. However, it is common for farmers to use several different pesticides during the growing season. On average, nearly 5 pesticides are applied to fields in Europe each year, ranging to more than 20 different pesticides for a single crop and field.^44^ Moreover, many of these pesticide products come already as a combination of multiple compounds, either mixtures of pesticides or mixtures of pesticides and formulation byproducts, to have broader and more potent effects in crop protection. Spray plans are even designed to combine many different compounds, with different modes of action, as an effective measure to reduce the development of resistance in pests, weeds and pathogens.^45−47^

The environmental impact of pesticide mixtures is a well-established concern.^48−50^ Although it cannot be assumed that a mixture per se is more problematic than individual pesticides, the interactions between the compounds are potentially important and are currently overlooked. In general, the combined risk of environmental mixtures is driven by one or a few substances, and when substances cause effects through a common mode of action, the likelihood of interactive effects, such as antagonism and synergism, increases. In contrast, for substances with dissimilar modes of action, response addition is likely.^51^ Moreover, studies have demonstrated that at certain mixture levels, when both synergistic and antagonistic effects are present, these interactive effects may even cancel each other out, resulting in simple additivity.^52^ For example, studies have shown that azole fungicides can enhance the toxicity of pyrethroid insecticides in invertebrates due to enzyme inhibition,^53^ whereas some herbicide mixtures, such as glyphosate and atrazine, can exhibit antagonistic interactions that reduce individual toxic effects.^54^ There is an ongoing debate about the extent to which the risks of individual pesticides can be assessed additively, with evidence suggesting that the synergistic potential of effects needs to be considered.^55^ Although a considerable number of experiments have been conducted to study the effects of chemical mixtures, the majority of mixture experiments have been carried out involving only a few compounds.^56,57^ A systematic review showed that 62% of the experiments involved only binary mixtures and a further 18% involved ternary mixtures, whereas mixture experiments using the number of components that could be found in real agricultural systems were rare.^55^ However, the composition of these mixtures, specifically the number of compounds they contain, might be crucial for potential synergistic effects. While an earlier study focusing on 2–3 combinations identified very few such effects,^57^ a more recent study suggests that an increase in the number of combinations also increases the potential for synergistic impacts from pesticides.^58^

Such diverse pesticide mixtures are a particular concern for the exposure in soil systems, as up to 32 different residues could simultaneously be detected in agricultural soils.^10,59^ Recent research has focused on determining the risk quotient of such mixtures, revealing that pesticide residue mixtures pose a medium or high risk to soil organisms at 35% to 46% of investigated sites.^12,38,60,61^ It is thus essential to consider the cumulative and interactive effects of pesticide mixtures when assessing their impact on soil organisms and the functioning of agroecosystems.

### Application Frequency

While mixtures are already well recognized as a potential accelerator of pesticide stress, the application frequency has received far less attention in addressing the impact of pesticides on soil systems. In agricultural settings, pesticides are not applied in a single event but spread over the growing season. The frequency of pesticide applications depends again on pest pressure, crop type and management practices and can range from a few to multiple applications during a growing season. For example, the average frequency of pesticide applications in intensively treated crops, such as sugar beets and potatoes, ranges between 15 and 20 times per season.^62^

Currently, our understanding of the effects of pesticide application frequency on soil systems is limited. However, initial findings indicate that it could pose a critical risk to soils. For example, two studies have demonstrated that while single applications have only a limited and transient effect, repeated pesticide applications cause severe impact on soil systems.^63,64^ Similarly, simulations of spray plans indicate that repeated use of pesticides lead to thresholds for expected environmental risk being reached earlier.^65^ One possible explanation for the amplified impact is that under frequent use certain soil organisms fail to recover during the interval between applications. Indeed, most experimental studies revealing transient effects of pesticides indicate a recovery after application of 8–18 weeks.^29,35,36^ It is therefore questionable whether organisms can fully recover in the time between exposure events in a spray plan where an average field is subjected to more than one application per month.^66^ Investigating the role of application frequency for pesticide pressure in agricultural soils is therefore a crucial dimension that warrants further research.^16^

While current efforts in environmental risk assessment and ecotoxicological research primarily focus on the effect of the dosage of a given pesticide, there is limited understanding of how many different pesticides can be combined and how frequently a field can be treated within a season without putting the environment at risk.^64^ There is therefore an urgent need for a more holistic experimental effort, going beyond the one-dimensional assessment of dose responses and considering the complexity of pesticide mixtures and the temporal dimension of application frequencies. By gaining a deeper understanding of pesticide exposure in terms of dose, mixture, and frequency, we can provide better-informed recommendations for entire spray plans, similar to established dose guidelines. These insights will help to govern the pressure of pesticide applications on a given field across a season more effectively.

## Additional Considerations for More Realistic Exposure Scenarios

### Long-Term Exposure Effects

When considering pesticide pressure in agricultural soils, it is crucial to look beyond spray plans and the duration of a single cropping season. These application patterns endure year after year over decades, leading to long-lasting, continuous exposure to the applied pesticides with potential long-term effects. Currently, long-term effects have not received sufficient attention, as standard tests, which typically last between 2 to 56 days, do not permit the prediction of long-term effects.^67^ One approach to testing for more persistent effects using established ecotoxicological model organisms is through multigenerational or transgenerational studies. Such studies provided the first evidence that the long-term effects of pesticides harbor further potential hazards. Multigenerational studies, for example, have reported that earthworm reproduction decreased with increasing exposure time,^68,69^ or that the survival of springtails decreased upon pesticide exposure from one generation to the next.^70^ Furthermore, the effects of pesticides have been observed to persist for up to three generations after the last application in springtails,^71,72^ suggesting that pesticides can have a delayed long-term impact on a population.

However, in soil, exposure time scales are typically much longer than those observed in multigenerational studies because of the high persistence of pesticide residues. While some pesticides can degrade over time through microbial biodegradation, hydrolysis, photodegradation, and chemical transformation, complete degradation is not always achieved. Recent studies have brought attention to this persistence of pesticide residues, revealing mixtures of 2 to 17 different pesticide residues or transformation products in soils with no direct application for up to 20 years.^10,59,73,74^ Pesticide residues continuously enter the soil solution through various soil processes, including physical phenomena like thawing and freezing, biological activities such as bioturbation and feeding, as well as anthropogenic factors like soil management. These processes lead to the release of previously captured and bound pesticide residues into the soil solution,^75^ resulting in continuous exposure to a mixture of substances at very low dosages. A study by Gevao et al.^76^ showed that these soil-bound pesticides can be bioavailable to earthworms and, to a lesser extent, microorganisms. Although such low doses of pesticides can have a stimulating effect, meaning they induce a hormetic response, in individual groups of soil organisms,^77^ the chronic exposure to low concentrations of pollutants is generally deemed to adversely affect the fitness and viability of organisms, populations, or ecosystems.^78^ Furthermore, continuous exposure to low concentrations of pesticides can pose a currently underestimated long-term hazards, as their effects may not be immediately noticeable, creating a substantial legacy.^79^ The continuous exposure to low dosages of pesticide residues could further impede recovery, similarly to that discussed above for application frequency, as the exposure remains constant, varying only in dosage.^80^ The impact of these low pesticide concentrations is not yet fully understood, but their importance is undoubtedly overlooked in traditional dose–response studies.^81^ In addition, the effects of pesticide degradation products, which form and persist over time, pose an as yet poorly identified threat to nontarget organisms.^82^ In summary, there is strong evidence that longer time scales for pesticide exposure in agricultural soils must be considered to capture cumulative and delayed effects. Incorporating extended exposure durations into experimental research will provide a more realistic understanding of pesticide effects under field conditions and generate critical insights to improve environmental risk assessment scenarios.

### Realistic Environmental Exposure Scenarios

Moreover, realistic ecological conditions are needed, because traditional risk assessment and much current research test the effects of pesticides on specific organisms individually. However, soil systems are one of the most diverse ecosystems on Earth, hosting up to 57% of its biodiversity.^83^ Within these complex and interwoven communities, pesticides have the potential to harm not only individual organisms but also indirectly impact others. For instance, some organisms may be suppressed by pesticide exposure, while others proliferate in the vacant ecological niches.^84^ Schnug et al.^68^ found that exposure to pesticides altered the dominance structure of springtail communities, while Chelinho et al.^85^ observed reduced abundance and diversity for microarthropods, indicating shifts in community structure. Such changes can trigger cascading effects within the community, ultimately altering the stability of entire ecosystems.^28^ Furthermore, exposure to a toxicant may result in shortened life expectancy, reduced reproductive success, or altered behavior.^86^ These subtle, sublethal effects often manifest, particularly when interacting with other biotic entities.

There is a growing number of studies that have assessed the effects of pesticides at the community or ecosystem level, for example, community assessments of nematodes exposed to a fungicide that showed sensitive responses^87^ or the negative effects of pesticide cocktails observed in the community of arbuscular mycorrhizal fungi.^88^ Such experiments, however, are usually conducted under ideal growth, testing the impact of pesticides on nontarget organisms in isolation. This approach fails to account for the complex interactions between organisms, ecological guilds, and populations that make up functioning ecosystems,^86^ and hence represent a considerable oversimplification of real ecological conditions.^43^

### Additional Stressors under Agricultural Conditions

Under realistic conditions, soil organisms are confronted not only with other organisms but also with a multitude of anthropogenic and environmental stressors. Recent observations have emphasized that as the number of such stressors to the soil system increases, their combined effects become amplified and exceed the effects of any single factor alone, underlining the importance of synergistic effects in the presence of multiple stressors.^89−91^ Agricultural management is associated with several other stressors that may simultaneously act with pesticides in agricultural soils. The impact of management on agricultural land can lead to starvation due to the lack of organic inputs, compaction of soil structure from the use of heavy machinery, and contamination with other pollutants from excessive mineral fertilizer applications and the use of pharmaceuticals in veterinary practices imported by manure and slurry application.^92,93^ Several studies have indicated that such stressors interact critically with pesticide exposure. For example, it has been shown that the effects of pesticides on bumblebees are exacerbated when exposed to a combination of nutritional deprivation and pesticide exposure,^94^ or that the toxicity of pesticides to soil microbiota is increased in the presence of metal nanoparticles in soil,^95^ or that the nontarget effects of pesticides on soil microbial communities are altered in combination with fertilizers.^96^ In addition to management-related stressors, soil systems are increasingly challenged by future climate scenarios, characterized by more frequent heat waves, droughts, and flooding events,^90,91^ as well as their consequences, such as rising greenhouse gas concentrations and shifts in soil oxygen availability.^97^ These factors related to climate change also interact with pesticide pressure, compounding the increased challenges for soil organisms.^98^ The combination of pesticides with warming or drought, for instance, has been shown to increase their toxicity and prolong the recovery period of soil organisms, such as earthworms or springtails.^99,100^ When estimating the impacts of pesticides on soil systems, it is crucial to consider how pesticides will interact with other global change factors. The high number of potential stressor combinations presents a significant challenge for risk assessment, making it impossible to evaluate all possible interactions under all conditions. However, more experimental research simulating more realistic agroecological contexts will help highlight particularly critical combinations of stressors, which could ultimately help refine and improve ecological risk assessments. Failure to consider these other factors may increase the risk of overlooking pesticide effects that will have significant impacts under more realistic environmental conditions.

## Research Gaps and Future Research Needs

While pesticides are primarily tested for the effects at different dosages, the combined pesticide pressure resulting from mixtures and application frequency remains an underexplored factor in assessing pesticide risks, despite being critical for reflecting realistic agronomic pesticide application scenarios. Agroecosystem-based experimental studies should take the lead in addressing these gaps by evaluating pesticide effects under diverse abiotic conditions found in natural soil systems, allowing a more nuanced understanding of the indirect effects of pesticides and their interaction with global change factors such as climate and resource variability. Furthermore, more long-term exposure studies are essential, as pesticide exposure occurs season after season and persists beyond application due to the high persistence of pesticide residues in soils. By adopting such a holistic approach, applied agroecological research can provide insights that help refine environmental risk assessment to ensure that it captures long-term and system-level effects on the soil ensuring a more comprehensive evaluation of pesticide impacts on soil health. This shift in perspective is crucial for both the regulatory process and future research, ultimately leading to more effective pesticide management strategies that sustain soil health and ecosystem integrity.
